# The association between ambient temperature and sperm quality in Wuhan, China

**DOI:** 10.1186/s12940-020-00595-w

**Published:** 2020-04-28

**Authors:** Xiaochen Wang, Xiaojia Tian, Bo Ye, Yi Zhang, Xiaotong Zhang, Shichun Huang, Cunlu Li, Simin Wu, Rui Li, Yuliang Zou, Jingling Liao, Jing Yang, Lu Ma

**Affiliations:** 1 Department of Reproductive Medicine Center, Renmin Hospital, Wuhan University, Zhangzhidong Road (formerly Ziyang Road), Wuchang District, Wuhan, 430060 China; 2 Department of Healthcare Management, School of Health Sciences, Wuhan University, 115 Donghu Road, Wuchang District, Wuhan, 430071 China; 3 Centre for Diseases Control and Prevention of the Lianyungang Economic and Technological Development Area, No.28 Tianshan Road, Lianyun District, Lianyungang, 222000 China; 4 General Practice and Primary Care, Institute of Health & Wellbeing, University of Glasgow, General Practice & Primary Care, House 2 Room 303, 1 Horselethill Road, Glasgow, G12 9LX UK; 5 Global Health Institute, Wuhan University, 8 Donghunan Road, Wuchang District, Wuhan, 430072 China; 6 Department of Public Health, Medical College Wuhan University of Science and Technology, Huangjiahuxi Road, Hongshan District, Wuhan, 430065 China

**Keywords:** Ambient temperature, Sperm quality, Threshold effect

## Abstract

**Background:**

Few epidemiological investigations have focused on the influence of environmental temperature on human sperm quality. Here, we evaluated the potential association between ambient temperature and human sperm quality in Wuhan, China, and examined the interactive effect of particulate matter (PM_2.5_) and temperature.

**Methods:**

1780 males who had been living in Wuhan for no less than three months and received semen analysis at the Department of Reproductive Medicine in Renmin Hospital of Wuhan University between April 8, 2013 and June 30, 2015 were recruited. Daily mean meteorological data and air pollution data (PM_2.5_, O_3_ and NO_2_) in Wuhan between 2013 and 2015 were collected. A generalized linear model was used to explore the associations between ambient temperature and sperm quality (including sperm concentration, percentage of normal sperm morphology, and progressive motility) at 0–9, 10–14, 15–69, 70–90, and 0–90 days before semen examination, and the interaction between temperature and PM_2.5_.

**Results:**

The associations between ambient temperature and sperm quality were an inverted U-shape at five exposure windows, except for a lag of 0–9 days for sperm concentration. A 1 °C increase in ambient temperature above the thresholds was associated with a 2.038 (1.292 ~ 2.783), 1.814 (1.217 ~ 2.411), 1.458 (1.138 ~ 1.777), 0.934(0.617 ~ 1.251) and 1.604 (1.258 ~ 1.951) decrease in the percentage of normal sperm morphology at lag 0–9, lag 10–14, lag 15–69, lag 70–90, and lag 0–90 days, respectively. The interaction *p-*values of PM_2.5_ and temperature were mostly less than 0.05 at five exposure windows. When ambient temperature exposure levels were above the thresholds, a 0.979 (0.659–1.299) and 3.559 (0.251 ~ 6.867) decrease in percentage of normal sperm morphology per 1 °C increase in temperature at lag 0–90 days was observed in the PM_2.5_ ≤ *P*_50_ group and PM_2.5_ > *P*_50_ group, respectively.

**Conclusions:**

Our results indicate that exposure to ambient temperature has a threshold effect on sperm quality, and PM_2.5_ enhances the effect of temperature on sperm quality when temperatures are above the threshold.

## Background

In recent decades, a global decline of human sperm quality has been noted that includes low sperm production, inferior morphology, and poor motility [1, 2]. Male factors known to be involved in infertility account for about 40% of infertility cases [3], but the causes of male infertility are multiple and complex. Environmental exposure may have an important impact on human sperm quality [4, 5], and some studies have examined the effect of ambient air pollution on semen quality [6]. For example, Wu et al. investigated semen from 1759 men in Wuhan, China between 2013 and 2015, and the results suggested that exposure to ambient particulate matter (PM) adversely affects semen quality during sperm development [4]. However, ambient temperature as an important environmental factor has not drawn much public health attention for its impact on sperm development [7, 8].

Climate issues have been associated with various subclinical and clinical health problems [9–12], and some studies in animal models show that an increased testis temperature of approximate 1.5 °C reduces sperm production and increase abnormalities during spermiogenesis [13, 14]. However, the epidemiology of the influence of environmental temperature on sperm quality has not been well studied. Although a retrospective cohort study was conducted in Italy and suggested an effect of environmental temperature on sperm quantity, confounding variables such as demographic characteristics were not well controlled [15]. Similar results have been reported in 2018, a study using a big-data approach has shown that both maximum and minimum temperatures in the day of collection were negative related to semen parameters, and the relationship were also confirmed in the 30 and 60 days before collection, but not in the 90 days before collection [16]. Meanwhile, the opposite finding has been reported, Momen et al. performed a prospective study and reported that semen parameters were also within normozoospermic levels when under high environmental temperature [17]. The environmental temperature varies geographically, so studies of the impact of environmental temperature on sperm quality should be conducted in various areas. Wuhan, which is the capital of Hubei province in Central China, is one of the “Three Furnace-like Cities” along the Yangtze River due to its extremely high temperatures in the summertime. The unique climate characteristics of Wuhan make this city a suitable area for studying the effect of environmental temperature on sperm quality.

Spermatogenesis takes approximately 90 days and includes three key periods: 0–9, 10–14, and 70–90 days before semen ejaculation, which correspond to sperm storage in the epididymis, sperm motility development, and spermatogenesis, respectively [18], and lag15–69 days was also involved to ensure the continuity. It is thus necessary to use five exposure windows, which are 0–9, 10–14, 15–69, 70–90, and 0–90 days, to investigate potential adverse effects of environmental factors on sperm quality in each key period of sperm development [19, 20]. Lafuente and colleagues proposed that air pollution could affect sperm functionality in the late phases of spermatogenesis in their systematic review on air pollution and sperm quality [21], but there has been no research that explores the stages of spermatogenesis in which ambient temperature exposure affects sperm quality.

In the present study, we recruited 1780 adult men who received semen analysis at the Department of Reproductive Medicine in Renmin Hospital of Wuhan University to analyze the potential association between ambient temperature and sperm quality overall and during each key stage of spermatogenesis. Furthermore, the interactive effect of PM_2.5_ and temperature on sperm quality were examined as well.

## Methods

### Study population

**Fig. 1:**
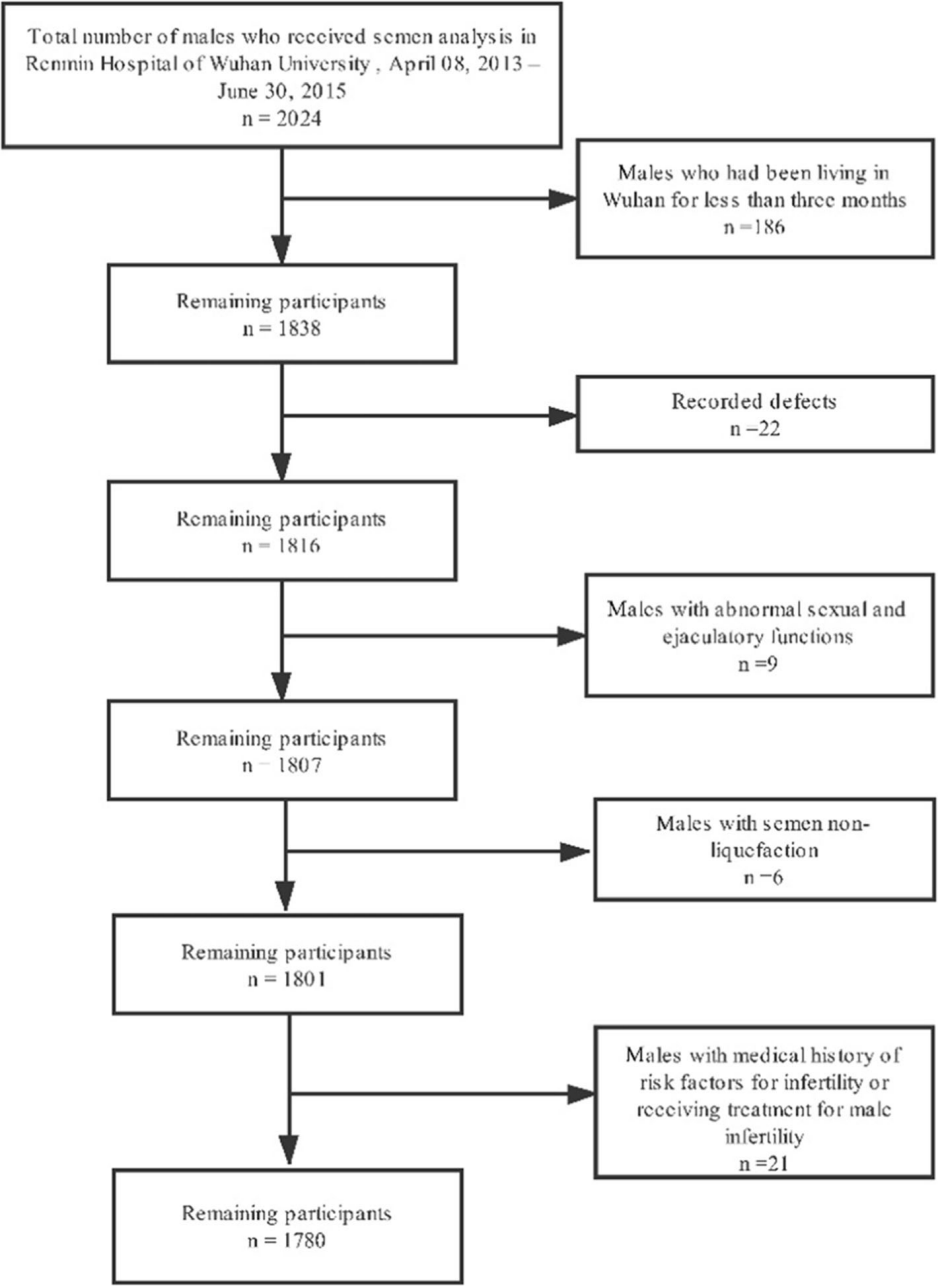
Flow chart of the study population selection

Between April 08, 2013 and June 30, 2015, a total of 2024 males attended the Department of Reproductive Medicine in Renmin Hospital of Wuhan University for semen analysis. The men attended to our reproductive medicine central contained two parts: men who undergo routine physical examinations and part of an infertile couple. The participants in our research were screened from them, and the flow chart of the study population selection was shown in Fig. 1. The total of 1838 men who had been living in Wuhan for no less than 3 months were recruited. We excluded 22 men whose records had defects, 9 men who had abnormal sexual and ejaculatory functions, 6 men whose semen were not able to be liquefied and 21 men who had medical history of risk factors for infertility or receiving treatment for male infertility. Finally, the total of 1780 men was included as the study participants. The individual characteristics (age, height, weight, smoking status, educational level, etc.), season of semen sample collection, and days of abstinence came from the electronic medical record. The study was approved by the ethics committee of Wuhan University, and all of the data included in the analysis were anonymized.

### Semen analysis

All of the participants were instructed to abstain from ejaculation for 2 to 7 days before providing semen samples. All of the samples were stored in sterile glass containers, and analyses were performed within 1 h after collection. To control for potential bias caused by dehydration, different pH, and temperatures, semen samples were liquefied at 37 °C for 20 min before analysis.

The semen quality was analyzed by Computer Assisted Semen Analysis (CASA) (WLJY-9000; Beijing Weili New Century Science & Tech Dev Co, Ltd., Beijing, China). The semen quality parameters included sperm concentration, percentage of normal sperm morphology, and progressive motility. All of the analyses were performed according to WHO standardized protocols [22]. Quality control and proficiency testing were carried out routinely by the laboratory technicians.

### Environmental data

We obtained the meteorological data from the China National Meteorological Information Centre (http://data.cma.cn/data/cdcindex/cid/6d1b5efbdcbf9a58.html) for the study period in Wuhan, including daily average relative humidity and daily average temperature. The data were presented as 24-h averages, and all participants in Wuhan had the same assigned daily mean value of average temperature and relative humidity. Daily mean air pollution (PM_2.5_, O_3_ and NO_2_) air quality indices (AQIs) were collected from the Wuhan Environmental Protection Bureau (http://hbj.wuhan.gov.cn/viewAirDarlyForestWaterInfo.jspx) and there were 10 national air quality monitoring stations in Wuhan covering the entire study period, PM_2.5_ and NO_2_ were presented as 24-h averages, O_3_ was presented as 8-h averages. The AQIs were converted to concentrations in μg/m^3^ according to the Technical Regulation on Ambient Air Quality Index [23]. For each day, we calculated the average concentration of 10 monitoring sites as daily mean air pollution exposures of all participants in Wuhan.

We employed five exposure windows to estimate the effects of ambient temperature on sperm quality at the key stages of sperm development (0–9, 10–14, 15–69, 70–90, and 0–90 days before semen ejaculation, respectively). The individual exposure levels (temperature, relative humidity, and PM_2.5_, O_3_ and NO_2_) of subjects were estimated by taking the average of each variable at corresponding exposure windows.

### Statistical analysis

Descriptive analysis was performed on environmental data and sperm parameters at the five windows of exposure (lag 0–9, lag 10–14, lag 15–69, lag 70–90, and lag 0–90 days). We applied a generalized linear model (GLM) to explore the associations between environmental temperature and sperm quality (including sperm concentration, percentage of normal sperm morphology, and progressive motility) at five exposure windows. Models were adjusted for body mass index (BMI) (< 18.5, 18.5 ~ 25, > 25), age (< 30, 30–34, 35–39, ≥40), education level (less than secondary education, secondary education, higher than secondary education), smoking status (non-smokers, smokers), season of semen sample collection (spring, summer, autumn, winter), days of abstinence (2 ~ 3, 4 ~ 5, 6 ~ 7), and relative humidity. To determine the threshold of temperature, we first used a generalized linear model with natural cubic splines to fit exposure-response curves between temperature and sperm quality. Because the difficulties of arbitrariness by researchers in selecting the number of degrees of freedom (df) [24], the selection of df of ambient temperature and relative humidity were based on previous studies [25, 26], df for temperature and relative humidity at each exposure windows were selected as 3. Then we found the exposure-response curves had inverse U-shapes or linear. When the curve was inverted U-shaped, the temperature at highest point of the U-shaped was chosen as the threshold, when the curve was linear, there was no threshold.

The study population was classified into a low temperature group and high temperature group by threshold values to assess the impact of cold and heat temperature on sperm quality. Sensitivity analysis was conducted by adding the PM_2.5_, O_3_ and NO_2_ to the model to evaluate the robustness of the results. We also performed analysis in a subgroup (*n* = 792) that excluded subjects with abnormal sperm concentration (< 15 × 10^6^/ml), percentage of normal sperm morphology (< 4%), and progressive motility (< 32%) according to the WHO reference levels [20]. The interactive effect of PM_2.5_ and temperature was tested by incorporating a multiplicative interaction term in the model. The interactive effect was considered statistically significant if the interaction term *p*-value was less than 0.05. To evaluate the specific modification of PM_2.5_, low and high temperature groups were divided into PM_2.5_ ≤ P_50_ and PM_2.5_ > P_50_ group by the 50th percentile of PM_2.5_, respectively, and the GLM was used to evaluate the effect of temperature on sperm quality in the four groups.

Results are reported as regression coefficients with 95% confidence intervals (*CI*) in sperm concentration, percentage of normal sperm morphology, and progressive motility per 1 °C increase in mean temperature and 1 μg/m^3^ increase of PM_2.5_. All statistical analyses were performed using SPSS statistical software (version 20.0) and R statistical software (version 3.5.1). All *p*-values were two-sided.

## Results

The descriptive analysis of socio-demographic characteristics of 1780 participants showed that the mean (±*SD*) age, body mass Index (BMI), sperm concentration, percentage of normal sperm morphology, and progressive motility of all participants were 33.5 ± 5.1 years old, 23.7 ± 3.3 kg/m^2^, 76.3 ± 50.2 mln/ml, 20.7 ± 19.9%, and 29.6 ± 16.4%, respectively (Table 1). More than half of the subjects were non-smokers (63.4%), and the sperm quality of non-smokers was higher than that of smokers. Semen collected in the spring or autumn had the lowest sperm quality. Abstinence time was positively associated with sperm concentration and the percentage of morphologically normal sperm.

**Table 1:** Demographic characteristics of the study participants (*N* = 1780)

Characteristics	N (%)	Mean ± SD		
		Sperm concentration (mln/ml)	Percentage of normal sperm morphology (%)	Progressive motility (%)
Body mass index (kg/m^2^)				
< 18.5	73(4.1)	79.3 ± 49.1	19.5 ± 17.7	28.1 ± 14.2
18.5 ~ 25	1146(64.4)	77.0 ± 52.1	20.9 ± 20.0	29.4 ± 16.7
> 25	561(31.5)	74.6 ± 46.2	20.5 ± 19.9	30.1 ± 16.1
Age (years)				
< 30	363(20.4)	76.3 ± 57.9	19.1 ± 19.2	30.4 ± 16.1
30–34	759(42.6)	76.53 ± 49.4	20.4 ± 19.4	30.1 ± 16.8
35–39	444(24.9)	75.0 ± 47.2	21.3 ± 20.2	28.3 ± 16.1
≥ 40	214(12.1)	78.2 ± 44.3	23.1 ± 21.9	28.5 ± 15.8
Education				
Less than secondary education	498(28.0)	73.7 ± 49.8	19.8 ± 19.2	29.2 ± 16.7
Secondary education	686(38.5)	76.5 ± 47.6	21.7 ± 20.3	29.0 ± 15.8
College and higher	596(33.5)	78.2 ± 53.1	20.2 ± 19.8	30.4 ± 16.6
Smoking				
Non-smokers	1129(63.4)	77.6 ± 51.2	20.7 ± 19.9	29.9 ± 16.6
Smokers	651(36.6)	74.0 ± 48.1	20.6 ± 19.9	28.8 ± 15.9
Season				
Spring	530(29.8)	70.4 ± 43.3	27.3 ± 24.2	29.2 ± 15.1
Summer	487(27.4)	74.4 ± 47.9	19.0 ± 18.4	29.6 ± 17.1
Autumn	448(25.1)	82.6 ± 59.3	14.4 ± 12.2	29.2 ± 16.2
Winter	315(17.7)	80.1 ± 48.7	20.9 ± 19.6	30.4 ± 17.4
Days abstaining (days)				
2 ~ 3	307(17.2)	74.79 ± 47.32	17.38 ± 16.86	29.99 ± 16.84
4 ~ 5	1009(56.7)	75.57 ± 53.17	19.65 ± 18.96	28.77 ± 16.38
6 ~ 7	464(26.1)	78.95 ± 45.03	25.19 ± 22.91	30.95 ± 16.04

The mean relative humidity for each of the five exposure windows was similar, but there was greater variability in mean temperature and concentration of PM_2.5_. The average exposure levels of mean temperature were 17.50 ± 8.60 °C, 17.41 ± 8.67 °C, 17.47 ± 7.95 °C, 16.27 ± 8.70 °C, and 16.87 ± 7.55 °C for lag 0–9, lag 10–14, lag 15–69, lag 70–90, and lag 0–90 days, respectively, while the concentration of PM_2.5_ was the highest at exposure window of lag 70–90 days, and the mean value was 93.91 ± 44.40 μg/m^3^ (Table 2).

**Table 2:** Summary statistics of PM_2.5_ levels and meteorological data by exposure period

	Mean ± SD	min	*P*_25_	*P*_*50*_	*P*_75_	max
PM_2.5_ (μg/m^3^)						
0–9	82.17 ± 39.97	26.17	54.11	74.17	96.92	206.52
10–14	82.68 ± 43.91	24.22	52.34	72.32	99.16	269.00
15–69	85.94 ± 38.16	32.13	57.40	77.88	103.79	178.81
70–90	93.91 ± 44.40	29.86	56.69	87.42	117.10	214.45
0–90	87.19 ± 34.70	37.26	61.08	78.09	110.21	161.53
Relative humidity (%)						
0–9	78.27 ± 6.18	56.90	74.50	78.80	82.90	90.00
10–14	78.18 ± 7.44	54.40	73.00	78.60	83.80	93.20
15–69	77.69 ± 3.30	69.40	74.93	78.00	79.85	85.38
70–90	78.22 ± 4.88	61.90	75.08	78.95	81.69	88.57
0–90	78.12 ± 2.45	73.70	76.24	77.64	79.74	84.10
Mean temperature (°C)						
0–9	17.50 ± 8.60	0.54	9.49	18.73	24.76	32.57
10–14	17.41 ± 8.67	−0.20	10.09	18.71	24.61	32.94
15–69	17.47 ± 7.95	4.03	10.40	18.51	24.28	30.94
70–90	16.27 ± 8.70	2.59	7.85	16.59	24.01	32.26
0–90	16.87 ± 7.55	4.80	9.87	17.15	23.82	29.22

Abbreviations: *min* minimum, *P*_25_: 25th percentile, *P*_*50*_: median, *P*_75_: 75th percentile, *max* maximum, *SD* standard deviation, *PM*_*2.5*_ particulate matter ≤2.5 μm in aerodynamic diameter

**Fig. 2:**
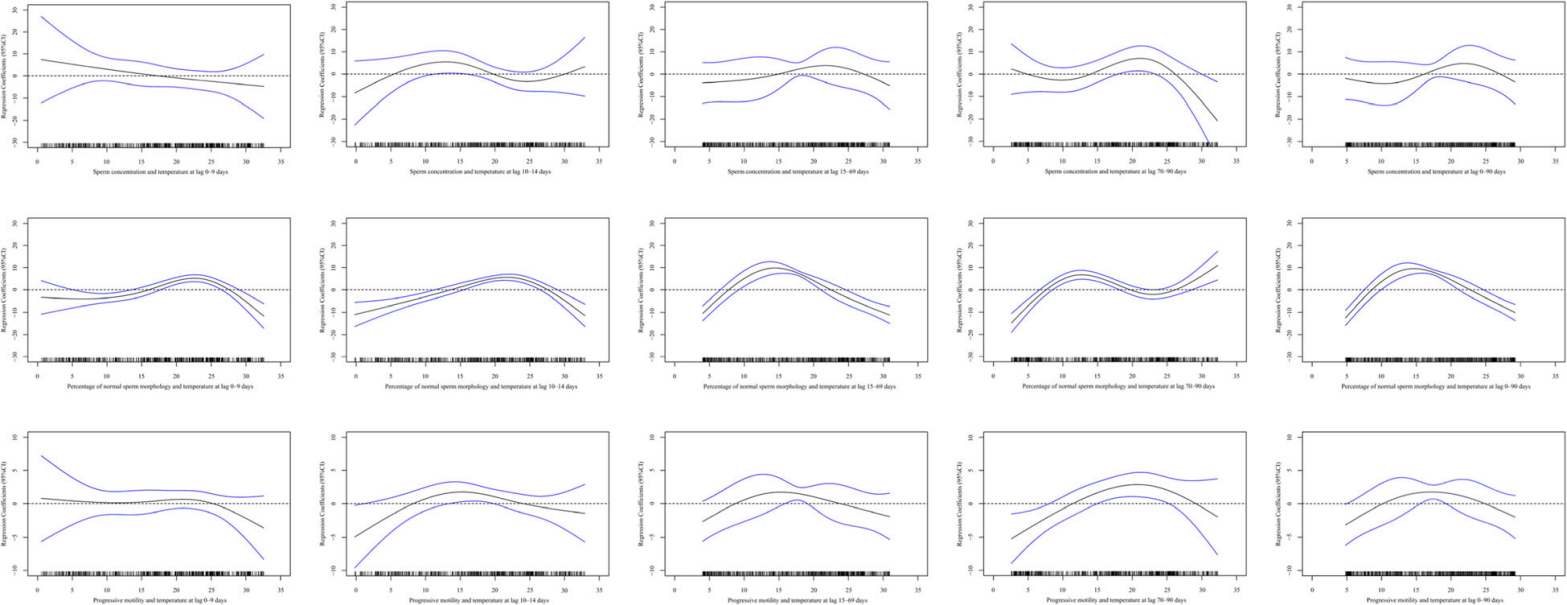
Exposure-response curves and 95% confidence intervals (*CI*s) for the relationship between environment temperature and sperm quality (sperm concentration, percentage of normal sperm morphology, and progressive motility) at four exposure windows (lag 0–9, lag 10–14, lag 15–69, lag 70–90 and lag 0–90 days) estimated using a generalized linear model by including a natural cubic spline function (df = 3) of temperature exposure adjusted for body mass index (BMI), education level, smoking status, season of semen sample, days abstaining, and relative humidity. The vertical black lines along the horizontal axes give the distribution of ambient temperature of the study population

The associations between the daily mean temperature and the percentage of normal sperm morphology and progressive motility were generally an inverted U-shape at the five exposure windows, indicating that threshold effects existed throughout the entire and at each of the key stages of spermatogenesis. The exposure-response curves of daily mean temperature and sperm concentration also had inverse U-shapes at lag 10–14, lag 15–69, lag 70–90, and lag 0–90 days, while the exposure-response curve was linear at lag 0–9 days (Fig. 2). The threshold values of temperature were 12.88 °C, 21.75 °C, 21.09 °C and 21.89 °C at lag 10–14, lag 15–69, lag 70–90, and lag 0–90 days for sperm concentration. The threshold values of temperature were 22.61 °C, 21.72 °C, 14.45 °C, 12.68 °C, and 14.69 °C at lag 0–9, lag 10–14, lag 15–69, lag 70–90, and lag 0–90 days for the percentage of normal sperm morphology. The threshold values of temperature were 20.94 °C, 15.24 °C, 15.20 °C, 20.81 °C and 17.15 °C at lag 0–9, lag 10–14, lag 15–69, lag 70–90, and lag 0–90 days for progressive motility (Table S1).

The study population was classified into low temperature and high temperature groups based on the threshold values at the five exposure windows. And the regression coefficients and 95% confidence intervals (95% *CI*) of sperm quality in the GLM model are shown in Table 3. A statistically significant relationship was not found between ambient temperature and sperm concentration, with an exception at lag 70–90 days when the exposure temperature exceeded the threshold. In addition, progressive motility was associated with ambient temperature at lag 15–69 days and lag 70–90 days, with a 1 °C increase in temperature below the thresholds increasing progressive motility by 0.532, while a 1 °C increase in temperature above the thresholds decreased progressive motility by 2.019 at lag 70–90 days. However, the percentage of normal sperm morphology was decreased with the ascent of temperature over the entire period and at each key stage of spermatogenesis when the exposure temperature was above the threshold. A 1 °C increase of temperature above the thresholds was associated with a 2.038 (1.292 ~ 2.783), 1.814 (1.217 ~ 2.411), 1.458 (1.138 ~ 1.777), 0.934(0.617 ~ 1.251) and 1.604 (1.258 ~ 1.951) decrease in the percentage of normal sperm morphology at lag 0–9, lag 10–14, lag 15–69, lag 70–90, and lag 0–90 days, respectively. When males were exposed to temperature below the threshold levels at the five exposure windows, positive effects were observed. After controlling for the effect of PM_2.5_, O_3_ and NO_2_, the analysis yielded the similar results (Table S2, Table S3 and Table S4). When we performed an analysis in subgroup (*n* = 792) that excluded subjects with abnormal sperm concentration, percentage of normal sperm morphology, and progressive motility, the subgroup analysis yielded the results that the percentage of normal sperm morphology were consistent between “normal” group and whole group, and we did not find a significant association between temperature and sperm concentration (*P* > 0.05). However, sperm progressive motility was positive associated with ambient temperature in “normal” group (*P* < 0.05) (Table S5).

**Table 3:** Associations between mean temperature and sperm quality at five exposure windows

Lag (days)	Sperm concentration (mln/ml)		Percentage of normal sperm morphology (%)		Progressive motility (%)	
	*β* (95% *CI*) value	*P* value	*β* (95% *CI*) value	*P* value	*β* (95% *CI*) value	*P* value
0–9						
≤ Threshold	− 0.365(− 0.967,0.238)	0.236	0.750(0.432,1.067)	< 0.001	−0.113(− 0.420,0.195)	0.472
> Threshold			−2.038(−2.783,-1.292)	< 0.001	−0.507(−1.132,0.118)	0.112
10–14						
≤ Threshold	1.204(−0.291,2.699)	0.114	0.695(0.413,0.977)	< 0.001	0.299(−0.109,0.706)	0.151
> Threshold	−0.018(− 0.893,0.829)	0.965	− 1.814(− 2.411,-1.217)	< 0.001	− 0.176(− 0.483,0.130)	0.259
15–69						
≤ Threshold	0.415(− 0.358,1.187)	0.293	1.896(1.409,2.383)	< 0.001	0.407(0.046,0.767)	0.027
> Threshold	−0.589(−2.694,1.517)	0.584	−1.458(− 1.777,-1.138)	< 0.001	− 0.268(− 0.582,0.046)	0.094
70–90						
≤ Threshold	0.480(− 0.375,1.335)	0.271	1.891(1.252,2.530)	< 0.001	0.532(0.236,0.828)	< 0.001
> Threshold	−5.953(−10.126,-1.781)	0.005	−0.934(− 1.251,-0.617)	< 0.001	−2.019(−3.232,-0.807)	0.001
0–90						
≤ Threshold	−0.076(−1.002,0.850)	0.872	2.726(2.070,3.381)	< 0.001	0.193(−0.131,0.517)	0.242
> Threshold	−1.445(−3.676,0.785)	0.204	−1.604(− 1.951,-1.258)	< 0.001	− 0.323(− 0.676,0.030)	0.073

The regression coefficients and 95% confidence intervals (CIs) were estimated using a generalized linear model for the relationship between mean temperature and sperm quality, adjusted for body mass index (BMI), education level, smoking status, season of semen sample, days abstaining, and relative humidity. The regression coefficients show changes in sperm quality with a 1 °C increase in mean temperature

0–9, 10–14, 15–69, 70–90, and 0–90 represent the five exposure windows (0–9, 10–14, 15–69, 70–90, and 0–90 days before semen examination, respectively)

≤ Threshold, > Threshold: the study population was divided into ≤ Threshold and > Threshold groups by threshold values of temperature on sperm quality. The threshold values of temperature were 12.88 °C, 21.75 °C, 21.09 °C and 21.89 °C at lag 10–14, lag 15–69, lag 70–90, and lag 0–90 days for sperm concentration; 22.61 °C, 21.72 °C, 14.45 °C, 12.68 °C, and 14.69 °C for the percentage of normal sperm morphology, and 20.94 °C, 15.24 °C, 15.20 °C, 20.81 °C and 17.15 °C at lag 0–9, lag 10–14, lag 15–69, lag 70–90, and lag 0–90 days for progressive motility

We found evidence of an effect modification by PM_2.5_ on exposure to ambient temperatures through the entire period and at each key stage of spermatogenesis (interaction *p-*value < 0.05) with an exception at lag 10–14 days. Moreover, when ambient temperatures were above the thresholds, the group exposed to higher PM_2.5_ concentrations was more affected by ambient temperature (Table 4). For instance, when the temperature exposure levels were above the thresholds, a 0.979 (0.659–1.299) decrease in the percentage of normal sperm morphology was observed in the PM_2.5_ ≤ *P*_50_ group per 1 °C increase of ambient temperature at lag 0–90 days. In comparison, males among the PM_2.5_ > *P*_50_ group had a 3.559 (0.251 ~ 6.867) decrease in the percentage of normal sperm morphology per 1 °C increase of temperature at lag 0–90 days.

**Table 4:** Interaction effects of environmental PM_2.5_ and temperature on the percentage of normal sperm morphology (%) at five exposure windows

Lag (days)	PM_2.5_ ≤ *P*_50_		PM_2.5_ > *P*_50_		Interaction *P* values
	*β* (95% *CI*) value	*P* value	*β* (95% *CI*) value	*P* value	
0–9					
≤ Threshold	1.096(0.556,1.636)	< 0.001	0.258(−0.218,0.733)	0.288	0.038
> Threshold	− 2.740(− 3.711,-1.769)	< 0.001	−8.162(− 11.400,-4.924)	< 0.001	0.158
10–14					
≤ Threshold	0.936(0.341,1.531)	0.002	0.659(0.303,1.014)	< 0.001	0.537
> Threshold	− 2.009(− 2.698,-1.320)	< 0.001	− 5.549(− 7.527,-3.572)	< 0.001	0.734
15–59					
≤ Threshold	5.128(5.128,5.128)	< 0.001	1.900(1.412,2.388)	< 0.001	< 0.001
> Threshold	−1.135(− 1.438,-0.833)	< 0.001	1.772(−0.240,3.785)	0.084	< 0.001
70–90					
≤ Threshold	3.206(1.551,4.862)	< 0.001	2.561(1.793,3.329)	< 0.001	0.663
> Threshold	−0.554(− 0.939,-0.169)	0.005	−2.671(− 3.724,-1.619)	< 0.001	< 0.001
0–90					
≤ Threshold	− 5.457(− 7.607,-3.306)	< 0.001	2.822(2.170,3.474)	< 0.001	0.016
> Threshold	− 0.979(−1.299,-0.659)	< 0.001	−3.559(− 6.867,-0.251)	0.035	< 0.001

The regression coefficients and 95% confidence intervals (CIs) were estimated using a generalized linear model for the relationship between mean temperature and sperm quality, adjusted for body mass index (BMI), education level, smoking status, season of semen sample, days abstaining, and relative humidity. The regression coefficients show changes in the percentage of normal sperm morphology with a 1 °C increase in mean temperature. Interaction *p*-values: the interactive effect of PM_2.5_ and temperature on sperm quality was considered statistically significant if the interaction term *p*-value was less than 0.05

0–9, 10–14, 15–69, 70–90, and 0–90 represent the five exposure windows (0–9, 10–14, 15–69, 70–90, and 0–90 days before semen examination, respectively)

≤ Threshold, > Threshold: the study population was divided into ≤ Threshold and > Threshold groups by threshold values of temperature on sperm quality. The threshold values of temperature were 22.61 °C, 21.72 °C, 14.45 °C, 12.68 °C, and 14.69 °C at lag 0–9, lag 10–14, lag 15–69, lag 70–90, and lag 0–90 days for the percentage of normal sperm morphology

PM_2.5_ ≤ P_50_, PM_2.5_ > P_50_: the study population was divided into PM_2.5_ ≤ P_50_ and PM_2.5_ > P_50_ groups by the 50th percentile of PM_2.5_. PM_2.5_: particulate matter ≤2.5 μm in aerodynamic diameter

## Discussion

In this study, we examined the association between environmental temperature and sperm quality among 1780 men from one hospital in Wuhan. A threshold effect of ambient exposure temperature on sperm quality was found. A decreased percentage of normal sperm morphology was associated with the increase of ambient temperature above the thresholds at five exposure windows, whereas the effect of exposure temperature below the thresholds was significantly positive. However, a statistically significant association between ambient temperature and sperm concentration or progressive motility only appeared at the early stages of sperm development (lag 15–69 days or lag 70–90 days). And we observed that PM_2.5_ enhanced the effect of ambient temperature on sperm quality when exposure temperatures were above the thresholds.

Seasonal differences have been found for semen quality in previous studies [19, 27], with improved sperm quality during winter and spring. In our study, we found that the population in Wuhan had the best sperm quality in autumn and spring. As temperature change is a main feature of the four seasons, this led us to question whether ambient temperature is the main factor for this seasonal change pattern. Laboratory data from animal models indicates that physiological temperature is an important cause of poor sperm quality [14]. However, few studies addressed environmental temperature; one study conducted in Italy found a significant relationship between environmental temperature and sperm concentration at lag 3 months, indicating that the environmental temperature had an adverse effect on sperm quality [15]. While Momen et al. reported the opposite finding that semen parameters were within normozoospermic levels when under high environmental temperature [17]. Although our results found that daily mean temperature correlated with sperm concentration and progressive motility only at the early stage of sperm development (lag 15–69 days or lag 70–90 days), the correlations between ambient temperature and the percentage of normal sperm morphology at the five exposure windows were statistically significant.

The exposure–response curve between environmental temperature and health outcomes has been demonstrated in many districts around the world, and the relationships generally have been described as U-, V-, W-, or J-shapes [11, 28, 29]. In this study, a threshold effect of temperature on health outcomes was observed, and the exposure-response curves of ambient temperature and sperm quality had inverse U-shapes. Our results referred that the threshold values of five exposure windows were inconsistent, and the study population in ≤ threshold and > threshold group of five exposure windows were different. Therefore, the effect at lag 0–90 days in two groups were not simply equal to the sum of the former four estimates at lags 0–9, 10–14, 15–69 and 70–90 days. Our results indicated that ambient temperature only at 70–90 days prior to semen ejaculation was associated with sperm concentration. Consistent with this finding, a study in Wuhan has reported that PM_2.5_ exposure only at lag 70–90 days is significantly associated with sperm concentration [4]. Both findings suggest that ambient factors tend to affect sperm concentration and sperm progressive motility at the early stages of sperm development, whereas ambient temperature affects the percentage of normal sperm morphology through all stages of spermatogenesis.

Many research groups have attempted to elucidate the underlying mechanisms of the association between ambient temperature and sperm quality, but they remain unclear. He and colleagues proposed that hot days could cause physiological stress [30], and Cheng et al. utilized an animal model to show that the excessive production of ROS induced by high temperature could disrupt the integrity of DNA in sperm cells and in turn decrease sperm quality [31]. Wang and colleagues have shown that high temperatures can cause apoptosis of spermatogenic cells by overexpression of heat shock proteins (HSP), which induces spermatogenic disorders [32]. Further investigations are required to clarify the detailed underlying biological mechanisms of the effects of ambient temperature on sperm quality.

Previous studies have demonstrated that ambient temperature and air pollution may interact synergistically to affect health outcomes [33–35]. Kim and colleagues found that temperature modified the effect of PM_10_ and increased the risk of daily mortality [33]. An influence of air pollution on sperm quality has been reported in several studies [36, 37], and it has been reported that air pollution is correlated with temperature [38]. Altogether, ambient temperature and air pollution may interact to affect sperm quality. However, no study has explored the modification effect of air pollution for temperature on sperm quality so far. In our study, We found that PM_2.5_ enhanced the temperature effect on sperm quality when exposure temperatures reached high levels. Some laboratrory data suggest that PM_2.5_ inhalation can cause inflammation and oxidative stress [39, 40]. Since both PM_2.5_ and high ambient temperatures can cause oxidative stress, it is rational to think that there is an interactive effect between them.

One strength of this study is that we are the first to investigate the nonlinear relationship between ambient temperature and sperm quality, finding that daily mean temperature has a threshold effect on sperm quality. In addition, our analysis controlled for various potential confounders, including body mass index (BMI), age, education level, smoking status, season of semen sample collection, days of abstinence, and relative humidity. Moreover, we took the effect of PM_2.5_ into account and examined whether there was an interaction between daily mean temperature and PM_2.5_ on sperm quality.

However, there are several limitations in current study. Despite the participants were selected through several inclusion and exclusion criteria, the subjects from hospital could not fully represent the general population, this might cause a misestimate of the association between ambient temperature and semen quality. It need to be explored in further study whether there are differences in the association between temperature and semen parameters in “normal” and “abnormal” people. Secondly, the city-wide outdoor average temperatures were used instead of individual temperature exposures, lacking of indoor temperature data and activity patterns of participants might lead to exposure measurement error. More advanced techniques and methods, such as land use regression models, are required for more accurate individual exposure assessment. Thirdly, the variation in temperature over the day was not taken into consideration in this study, a future study may focus on the association between variation in temperature over the day and sperm quality, and the comparison of the effects of daily environmental temperature and variation in temperature.

## Conclusion

In conclusion, our study indicates that the exposure to ambient temperature has threshold effects on sperm quality, and there are significant interactive effects of temperature and PM_2.5_ on sperm quality. The effects of ambient temperature were more adverse at high levels of PM_2.5_ when temperatures were above the thresholds. These findings may have important implications for the association between temperature, PM_2.5_, and reproductive health.

## Supplementary information

**Additional file 1: Table S1.** The threshold values of temperature on sperm quality. **Table S2.** Sensitivity analysis of the association between mean temperature and sperm quality (adding PM_2.5_ into the model). **Table S3.** Sensitivity analysis of the association between mean temperature and sperm quality (adding O_3_ into the model). **Table S4.** Sensitivity analysis of the association between mean temperature and sperm quality (adding NO_2_ into the model). **Table S5.** Analysis in subgroup of the associations between mean temperature and sperm quality.

## Data Availability

Not applicable.
