# Supplementary material for: The association between ambient temperature and sperm quality in Wuhan, China
Source: Environ Health. 2020 Apr 28;19:44. doi: 10.1186/s12940-020-00595-w (PMC7189467; doi:10.1186/s12940-020-00595-w)
Supplement: Supplementary file 1 — Additional file 1: Table S1. The threshold values of temperature on sperm quality. Table S2. Sensitivity analysis of the association between mean temperature and sperm quality (adding PM2.5 into the model). Table S3. Sensitivity analysis of the association between mean temperature and sperm quality (adding O3 into the model). Table S4. Sensitivity analysis of the association between mean temperature and sperm quality (adding NO2 into the model). Table S5. Analysis in subgroup of the associations between mean temperature and sperm quality. [file 12940_2020_595_MOESM1_ESM.docx]

| Table S1. The threshold values of temperature on sperm quality (°C) | | | | |
| --- | --- | --- | --- | --- |
| Lag (days) | | Sperm concentration | Percentage of normal sperm morphology | Progressive motility |
| 0-9 |  | | 22.61 | 20.94 |
| 10-14 | 12.88 | | 21.72 | 15.24 |
| 15-69 | 21.75 | | 14.45 | 15.20 |
| 70-90 | 21.09 | | 12.68 | 20.81 |
| 0-90 | 21.89 | | 14.69 | 17.15 |

| Table S2. Sensitivity analysis of the association between mean temperature and sperm quality (adding PM_2.5_ into the model) | | | | | | | | |
| --- | --- | --- | --- | --- | --- | --- | --- | --- |
| Lag (days) | Sperm concentration (mln/ml) | |  | Percentage of normal sperm morphology (%) | |  | Progressive motility (%) | |
|  | *β* (95% *CI*) value | *P* value |  | *β* (95% *CI*) value | *P* value |  | *β* (95% *CI*) value | *P* value |
| 0-9 |  |  |  |  |  |  |  |  |
| ≤Threshold | -0.828(-1.566,-0.090) | 0.028 | | 1.154(0.802,1.506) | <0.001 |  | -0.324(-0.650,0.003) | 0.052 |
| > Threshold |  |  |  | -3.204(-4.211,-2.197) | <0.001 |  | -0.171(-1.006,0.664) | 0.688 |
| 10-14 |  |  |  |  |  |  |  |  |
| ≤Threshold | -0.104(-1.735,1.526) | 0.900 |  | 0.964(0.654,1.274) | <0.001 |  | -0.141(-0.571,0.289) | 0.520 |
| > Threshold | 0.174(-0.876,1.225) | 0.745 |  | -2.436(-3.137,-1.736) | <0.001 |  | -0.158(-0.548,0.232) | 0.427 |
| 15-69 |  |  |  |  |  |  |  |  |
| ≤Threshold | -1.198(-2.627,0.230) | 0.100 |  | 2.989(2.163,3.814) | <0.001 |  | -1.176(-1.788,-0.565) | <0.001 |
| > Threshold | 0.514(-1.966,2.995) | 0.684 |  | -1.031(-1.481,-0.580) | <0.001 |  | -0.407(-0.853,0.038) | 0.073 |
| 70-90 |  |  |  |  |  |  |  |  |
| ≤Threshold | -0.369(-1.522,0.784) | 0.530 |  | 2.041(1.124,2.959) | <0.001 |  | 0.272(-0.125,0.669) | 0.180 |
| > Threshold | -6.783(-11.206,-2.360) | 0.003 |  | -1.147(-1.569,-0.725) | <0.001 |  | -2.306(-3.582,-1.030) | <0.001 |
| 0-90 |  |  |  |  |  |  |  |  |
| ≤Threshold | -2.958(-4.763,-1.154) | 0.001 |  | 4.097(3.072,5.123) | <0.001 |  | -1.745(-2.358,-1.132) | <0.001 |
| > Threshold | -0.621(-3.417,2.176) | 0.664 |  | -0.675(-1.242,-0.108) | <0.001 |  | -0.448(-1.067,0.172) | 0.156 |

The regression coefficients and 95% confidence intervals (*CI*s) were estimated using a generalized linear model for the relationship between mean temperature and sperm quality, adjusted for body mass index (BMI), education level, smoking status, season of semen sample, days abstaining, relative humidity, and PM_2.5_. The regression coefficients show the changes in sperm quality with a 1°C increase in mean temperature.

0-9, 10-14, 15–69, 70-90, and 0-90 represent the five exposure windows (0–9, 10–14, 15–69, 70–90, and 0–90 days before semen examination, respectively).

≤ Threshold, > Threshold: the study population was divided into ≤ Threshold and > Threshold groups by threshold values of temperature on sperm quality.

| Table S3. Sensitivity analysis of the association between mean temperature and sperm quality (adding O_3_ into the model) | | | | | | | | |
| --- | --- | --- | --- | --- | --- | --- | --- | --- |
| Lag (days) | Sperm concentration (mln/ml) | |  | Percentage of normal sperm morphology (%) | |  | Progressive motility (%) |  |
|  | *β* (95% *CI*) value | *P* value |  | *β* (95% *CI*) value | *P* value |  | *β* (95% *CI*) value | *P* value |
| 0-9 |  |  |  |  |  |  |  |  |
| ≤Threshold | -0.091(-0.760,0.578) | 0.790 |  | 0.190(-0.251,0.630) | 0.399 |  | -0.191(-0.653,0.271) | 0.418 |
| > Threshold |  |  |  | -2.131(-2.907,-1.354) | <0.001 |  | -0.535(-1.177,0.107) | 0.103 |
| 10-14 |  |  |  |  |  |  |  |  |
| ≤Threshold | 1.379(-0.113,2.872) | 0.070 |  | 0.551(0.224,0.878) | 0.001 |  | 0.329(-0.098,0.755) | 0.131 |
| > Threshold | 0.006(-0.805,0.817) | 0.988 |  | -1.973(-2.574,-1.372) | <0.001 |  | -0.194(-0.501,0.113) | 0.216 |
| 15-69 |  |  |  |  |  |  |  |  |
| ≤Threshold | 0.391(-1.126,1.908) | 0.613 |  | 3.642(2.980,4.304) | <0.001 |  | 0.267(-0.270,0.804) | 0.330 |
| > Threshold | -0.479(-2.674,1.715) | 0.669 |  | -1.867(-2.270,-1.464) | <0.001 |  | -0.156(-0.553,0.241) | 0.441 |
| 70-90 |  |  |  |  |  |  |  |  |
| ≤Threshold | -0.255(-1.439,0.929) | 0.673 |  | 2.481(1.891,3.071) | <0.001 |  | 0.443(0.038,0.848) | 0.032 |
| > Threshold | -7.554(-12.018,-3.090) | 0.001 |  | -0.461(-0.818,-0.105) | 0.011 |  | -2.336(-3.625,-1.047) | <0.001 |
| 0-90 |  |  |  |  |  |  |  |  |
| ≤Threshold | -0.139(-2.182,1.903) | 0.894 |  | 6.596(5.584,7.608) | <0.001 |  | 0.074(-0.623,0.771) | 0.835 |
| > Threshold | -1.328(-3.593,0.937) | 0.250 |  | -1.397(-1.934,-0.860) | <0.001 |  | -0.349(-0.903,0.206) | 0.218 |

The regression coefficients and 95% confidence intervals (*CI*s) were estimated using a generalized linear model for the relationship between mean temperature and sperm quality, adjusted for body mass index (BMI), education level, smoking status, season of semen sample, days abstaining, relative humidity, and O_3_. The regression coefficients show the changes in sperm quality with a 1°C increase in mean temperature.

0-9, 10-14, 15–69, 70-90, and 0-90 represent the five exposure windows (0–9, 10–14, 15–69, 70–90, and 0–90 days before semen examination, respectively).

≤ Threshold, > Threshold: the study population was divided into ≤ Threshold and > Threshold groups by threshold values of temperature on sperm quality.

| Table S4. Sensitivity analysis of the association between mean temperature and sperm quality (adding NO_2_ into the model) | | | | | | | | |
| --- | --- | --- | --- | --- | --- | --- | --- | --- |
| Lag (days) | Sperm concentration (mln/ml) | |  | Percentage of normal sperm morphology (%) | |  | Progressive motility (%) | |
|  | *β* (95% *CI*) value | *P* value |  | *β* (95% *CI*) value | *P* value |  | *β* (95% *CI*) value | *P* value |
| 0-9 |  |  |  |  |  |  |  |  |
| ≤Threshold | -0.375(-1.032,0.282) | 0.264 |  | 0.844(0.533,1.155) | <0.001 |  | -0.107(-0.415,0.200) | 0.494 |
| > Threshold |  |  |  | -1.693(-2.991,-0.395) | 0.011 |  | 0.037(-0.896,0.970) | 0.938 |
| 10-14 |  |  |  |  |  |  |  |  |
| ≤Threshold | 1.129(-0.310,2.568) | 0.124 |  | 0.690(0.410,0.969) | <0.001 |  | 0.313(-0.095,0.720) | 0.132 |
| > Threshold | 0.114(-0.931,1.158) | 0.831 |  | -2.144(-2.960,-1.327) | <0.001 |  | -0.068(-0.455,0.320) | 0.732 |
| 15-69 |  |  |  |  |  |  |  |  |
| ≤Threshold | -0.090(-1.081,0.902) | 0.859 |  | 2.364(1.741,2.986) | <0.001 |  | -0.321(-0.784,0.142) | 0.174 |
| > Threshold | 0.692(-4.579,5.963) | 0.797 |  | -1.726(-2.322,-1.129) | <0.001 |  | -0.229(-0.827,0.369) | 0.453 |
| 70-90 |  |  |  |  |  |  |  |  |
| ≤Threshold | 0.352(-0.593,1.297) | 0.465 |  | 2.511(1.728,3.293) | <0.001 |  | 0.331(-0.005,0.666) | 0.054 |
| > Threshold | -4.777(-10.088,0.534) | 0.078 |  | -1.546(-1.923,-1.170) | <0.001 |  | -2.261(-3.604,-0.919) | 0.001 |
| 0-90 |  |  |  |  |  |  |  |  |
| ≤Threshold | -0.946(-2.235,0.342) | 0.150 |  | 3.079(2.267,3.891) | <0.001 |  | -0.518(-0.973,-0.062) | 0.026 |
| > Threshold | -0.002(-5.061,5.058) | 0.999 |  | -1.908(-2.571,-1.246) | <0.001 |  | 0.097(-0.644,0.839) | 0.797 |

The regression coefficients and 95% confidence intervals (*CI*s) were estimated using a generalized linear model for the relationship between mean temperature and sperm quality, adjusted for body mass index (BMI), education level, smoking status, season of semen sample, days abstaining, relative humidity, and NO_2_. The regression coefficients show the changes in sperm quality with a 1°C increase in mean temperature.

0-9, 10-14, 15–69, 70-90, and 0-90 represent the five exposure windows (0–9, 10–14, 15–69, 70–90, and 0–90 days before semen examination, respectively).

≤ Threshold, > Threshold: the study population was divided into ≤ Threshold and > Threshold groups by threshold values of temperature on sperm quality.

| Table S5. Analysis in subgroup of the associations between mean temperature and sperm quality | | | | | | | | |
| --- | --- | --- | --- | --- | --- | --- | --- | --- |
| Lag (days) | Sperm concentration (mln/ml) | |  | Percentage of normal sperm morphology (%) | |  | Progressive motility (%) | |
|  | *β* (95% *CI*) value | *P* value |  | *β* (95% *CI*) value | *P* value |  | *β* (95% *CI*) value | *P* value |
| 0-9 |  |  |  |  |  |  |  |  |
| ≤Threshold | 0.171(-0.800,1.143) | 0.729 |  | 0.589(0.105,1.073) | 0.017 |  | -0.141(-0.419,0.137) | 0.320 |
| > Threshold |  |  |  | -2.066(-3.304,-0.829) | 0.001 |  | 0.336(-0.164,0.836) | 0.188 |
| 10-14 |  |  |  |  |  |  |  |  |
| ≤Threshold | 0.412(-1.909,2.733) | 0.728 |  | 0.622(0.166,1.079) | 0.008 |  | -0.220(-0.619,0.179) | 0.281 |
| > Threshold | 0.804(-0.601,2.209) | 0.262 |  | -2.670(-3.650,-1.691) | <0.001 |  | 0.288(0.025,0.550) | 0.032 |
| 15-69 |  |  |  |  |  |  |  |  |
| ≤Threshold | 0.543(-0.682,1.768) | 0.385 |  | 2.479(1.706,3.253) | <0.001 |  | -0.011(-0.331,0.308) | 0.945 |
| > Threshold | -0.651(-5.658,4.355) | 0.799 |  | -1.677(-2.185,-1.168) | <0.001 |  | 0.359(0.092,0.627) | 0.008 |
| 70-90 |  |  |  |  |  |  |  |  |
| ≤Threshold | 0.892(-0.488,2.272) | 0.205 |  | 1.692(0.733,2.651) | 0.001 |  | 0.389(0.117,0.662) | 0.005 |
| > Threshold | -2.760(-10.412,4.893) | 0.480 |  | -1.331(-1.831,-0.831) | <0.001 |  | -0.346(-1.306,0.614) | 0.480 |
| 0-90 |  |  |  |  |  |  |  |  |
| ≤Threshold | 0.010(-1.495,1.515) | 0.990 |  | 3.383(2.381,4.384) | <0.001 |  | -0.058(-0.345,0.230) | 0.694 |
| > Threshold | 0.495(-3.752,4.742) | 0.819 |  | -1.830(-2.385,-1.275) | <0.001 |  | 0.380(0.084,0.677) | 0.012 |

The regression coefficients and 95% confidence intervals (*CI*s) were estimated using a generalized linear model for the relationship between mean temperature and sperm quality, adjusted for body mass index (BMI), education level, smoking status, season of semen sample, days abstaining, and relative humidity. The regression coefficients show the changes in sperm quality with a 1°C increase in mean temperature.

0-9, 10-14, 15–69, 70-90, and 0-90 represent the five exposure windows (0–9, 10–14, 15–69, 70–90, and 0–90 days before semen examination, respectively).

≤ Threshold, > Threshold: the study population was divided into ≤ Threshold and > Threshold groups by threshold values of temperature on sperm quality.

subgroup (n =792): excluded subjects with abnormal sperm concentration (<15×10^6^/ml), percentage of normal sperm morphology (<4%), and progressive motility (<32%) according to the WHO reference levels.
